# Introduction to State Estimation of High-Rate System Dynamics

**DOI:** 10.3390/s18010217

**Published:** 2018-01-13

**Authors:** Jonathan Hong, Simon Laflamme, Jacob Dodson, Bryan Joyce

**Affiliations:** 1 Applied Research Associates, Emerald Coast Division, Niceville, FL 32578, USA; 2 Department of Civil, Construction, and Environmental Engineering, Iowa State University, Ames, IA 50011, USA; laflamme@iastate.edu; 3 Air Force Research Laboratory, Munitions Directorate, Eglin Air Force Base, FL 32542, USA; jacob.dodson.2@us.af.mil; 4 Energy Technologies and Materials, University of Dayton Research Institution, Dayton, OH 45469, USA; bryan.joyce.ctr@us.af.mil

**Keywords:** adaptive observers, high-rate, state estimation, dynamics, structural health monitoring

## Abstract

Engineering systems experiencing high-rate dynamic events, including airbags, debris detection, and active blast protection systems, could benefit from real-time observability for enhanced performance. However, the task of high-rate state estimation is challenging, in particular for real-time applications where the rate of the observer’s convergence needs to be in the microsecond range. This paper identifies the challenges of state estimation of high-rate systems and discusses the fundamental characteristics of high-rate systems. A survey of applications and methods for estimators that have the potential to produce accurate estimations for a complex system experiencing highly dynamic events is presented. It is argued that adaptive observers are important to this research. In particular, adaptive data-driven observers are advantageous due to their adaptability and lack of dependence on the system model.

## 1. Introduction

High-rate dynamics are described as a dynamic response from a high-rate (<100 ms) and high-amplitude (acceleration > 100 $g_{n}$) event such as a blast or impact. Systems which experience high-rate dynamics may undergo rapid changes that could lead to loss of economic investment or human lives [1]. There are unique challenges associated with research of structural health monitoring for systems experiencing high-rate dynamics. A system subject to high-rate dynamic environments can often experience a sudden plastic deformation, and damage can extend to the structure, electronics, and/or sensors. Rapid state estimation for these systems can be used within a feedback loop to prevent further damage and complete failure [2]. For example, rapid detection is required in the deployment of a blast mitigation system or in adapting control decisions for a hypersonic vehicle following a ballistic impact. In the problem of high-rate dynamic systems, the state estimator needs to be fast as well as robust to large uncertainties, non-stationarities, and heavy disturbances. State estimation is required when the desired states cannot be directly measured [3]. The area of research has been pioneered by Wiener [4], which led to Kalman’s work [5,6]. Kalman adopted a practically structured approach, with applications found notably in the spacecrafts used in the Apollo and Polaris missions. Since then, optimal estimation research has substantially grown in popularity. In addition, advances in estimation and control theory, along with computer science, have enabled the development of observers with rapid convergence properties. Such observers have the potential to produce safer and smarter systems capable of responding to real-time events. In particular, the capability of an estimator to sense, analyze, and predict the heath of a system, while experiencing high-rate dynamic environments, could be invaluable for many different domains. However, this identification and adaptation task can only be conducted provided an in-time state estimation during the high-rate dynamic event, where high-rate is herein defined as the microsecond range.

The high-rate problem contains many complexities that can be summarized as having

- large uncertainties in the external loads;
- high levels of non-stationarities and heavy disturbances; and
- generated unmodeled dynamics from changes in system configuration.

A high-rate estimator must also be capable of handling these three main particularities that differentiate high-rate dynamic systems from other systems. State estimation is often required for high-rate systems because of the large levels of noise, uncertainties, and disturbances that contaminate the sensor outputs. However, the end goal of the state estimation could be different depending on the application. A popular application is damage or fault detection, in particular when a fast decision needs to be made to protect the integrity of the system, for instance a shutdown. Another application is in the adaption of physical models. For example, a different control law could be desirable for a damaged system. Whatever the application may be, state estimation is the first enabling step.

The objective of this paper is to analyze existing applications and methods of state estimation that could be useful in solving the high-rate state estimation problem for complex systems that may have nonlinear and time varying dynamics. Advantages and disadvantages of each method, with the focus on microsecond convergence rates, capable of producing accurate estimations will be surveyed. The system’s observability, or how well the system can be reconstructed from measurements, is a critical concept that underlies the mathematical constructs of state estimators. It is very difficult to formulate a fully observable model of a complex physical system with high-rate system dynamics [7]. Because the focus of this paper is on the high-rate estimators’ performance, we do no go into details about the observability of these complex systems. Here, attention to what aspects negatively impact convergence of estimators and the comparison between observers will give insight into how convergence rates between observers may compare. In cases when simplicity versus complexity of estimators (i.e., for computation, properties, implementation, etc.) is mentioned, the connection should be made to convergence rates in the sense that simplicity correlates to faster convergence rates. However, the performance of observers is application-specific, and the findings will be dependent on the type of scenario. The observers presented were found to be most applicable to high rate systems. The objective is to familiarize the reader with some key observer background, and gain some insight into advanced techniques to improve observer performance with respect to the high-rate state estimation problem.

The rest of the paper is organized as follows. Section 2 discusses applications where high-rate state estimation could be useful. Section 3 describes the specific challenges associated with state estimation of high-rate dynamics. Section 4 gives the background on general types of observers and their broad applications. Section 5 makes a case for adaptive observers and their application to high-rate dynamics. Section 6 concludes the paper.

## 2. Applications for High-Rate State Estimation

This section discusses engineering systems for which high-rate state estimation can be particularly useful. Examples include civil structures exposed to blast, automotive safety systems against collisions, debris strikes to space shuttles, and aerial vehicles.

### 2.1. Civil Structures Exposed to Blast

The increase in number of terrorist attacks on civilian and military structures is a growing concern. One must also consider accidental events such as gas leaks, vehicular collisions, and chemical mishaps, all having the potential to cause instantaneous, important changes to civil structures. These important events have the potential to jeopardize the structural integrity resulting in partial or total loss. Passive blast mitigation strategies have been developed to absorb some shock. These methods include friction elements, laminated windows, hardened concrete, etc. [8,9,10]. However, passive mitigation techniques are limited in performance. As an improvement, semi-active and active mitigation methods have been researched and discussed.

One example of active blast mitigation is proposed by Wadley et al. in [11]. The authors discussed using high-speed actuators to deploy pre-compressed cellular material when a blast wave is detected. Electromagnetic emission sensors are capable of detecting the blast milliseconds before the wave arrival, therefore allowing time for the actuators to react. Using this technique, the material is able to absorb the shock waves through momentum cancellation. This method offers a larger shock absorption potential over passive methods. Nevertheless, the method is not capable of adaptive actuation based on the blast dynamics. While the utilization of adaptive actuation may improve blast mitigation performance, the reliance on a control rule adds computational time, let alone the reaction time of the actuation. As an example, the time of arrival of a blast produced by a 10 kg Trinitrotoluene (TNT) fluctuates between 0.3 and 100 ms for a distance of 1 to 40 m, requiring the sensing, estimation, and actuation process to be less than the time of arrival. In this situation, a high-rate observer would be critical in enabling the technology.

### 2.2. Automotive Safety Systems against Collisions

Fatal car accidents are on the rise [12] and improvements in our current safety measures have potential to decrease this number significantly. Current research in this field is geared toward smarter safety systems. An example is seen in [13], where the authors proposed a stereovision-based sensing method using stereo cameras and intelligent algorithms. The system classifies the occupant to determine if a child is present or if one of the passengers is in an unsafe position before deploying airbags. Although airbags play an important role in saving lives [14], this method addresses the issue that airbag systems unfortunately do sometimes cause unnecessary or even fatal injuries [15]. The child detection aspect is rather simple in that it can be done at the moment the vehicle is started. However, the unsafe position calculation is more challenging due to the fact that the calculation has to be done as an accident is occurring, in a fraction of a second [16]. The authors use a thin plate spline algorithm to boast processing times of 960 ms for the entire operation. It follows that smart vehicle safety systems could benefit from a high-rate observer robust to the non-stationary behaviors of the human body. It may, similar to the blast mitigation technology, enable the integration of more complex and better performing feedback systems.

### 2.3. Space Shuttle and Aerial Vehicles Prone to In-Flight Anomalies

The loss of the Columbia space shuttle in 2003 has brought light to the destructive potential of debris strikes. During the launch phase, a piece of insulating foam broke off and impacted the leading edge of the left wing while traveling at a velocity of 705 m/s [17]. This caused a breach in the thermal protection system ultimately leading to the destruction during re-entry [18]. This catastrophe led to the research and development of the NASA Debris Radar (NDR). The NDR uses sophisticated methods to target and track debris during launch. It is also capable of automatic debris characterization assigning ballistic numbers to assess the material type, size, release location, and threat associated with the object [19]. However, the NDR is an offsite ground radar system. There are scenarios where ground intervention is not possible, for instance during communication delays or visibility limitations. Furthermore, the NDR only provides information during ascent. In the case of Columbia, the damage occurred during launch, but the loss occurred during re-entry. It follows that an onboard spacecraft structural health monitoring system could improve the in-time detectability of damage. Analogous to space shuttles, aerial vehicles often face the possibility of damage arising from impact with foreign objects including bird, hail, and lightning strikes. Any damage during flight can cause issues in dangerous navigational uncertainties. Because of the speed at which space shuttles and aerial vehicles travel and the rate at which damage may occur, a high-rate estimator has the potential to substantially improve safety through early detection of anomalies.

In addition, the aerospace industry is making advancements in hypersonic aerial vehicles that have the possibility of facing challenges similar to those of space shuttles. Hypersonic is defined as speeds of Mach 5 or greater [20]. Mach 5 at an altitude of 10,000 m and −50 ${}^{\circ }$C corresponds to 1.5 km/s (0.93 mi/s). In comparison, the average Boeing passenger aircraft cruises at 600 mph or about Mach 0.8. It follows that a hypersonic vehicle travels at speeds at least six times greater than conventional passenger airplanes. At these speeds, there are possibilities for problems to quickly turn catastrophic. For instance, the distance travelled at Mach 5 is equivalent to 1.5 m (4.9 ft) over 1 ms. A 1.5 MHz sampling rate is necessary to obtain a 1 mm (0.04 in) travel resolution, thus requiring a high-rate observer.

## 3. Challenges in State Estimation for Systems Experiencing High-Rate Dynamics

State estimation of high-rate dynamics offers unique challenges. By definition of high-rate, estimators applicable to this research area will require rapid convergence. Take the hypothetical example of a civil structure exposed to blast equipped with an actively controlled protection system. The large uncertainties in the external loads appears in the form of lack of knowledge on the blast. The time, location, and size of the blast are all unknown. When a structure experiences a blast, it is likely to experience some damages which amount to non-stationarities and heavy disturbances. Structural damage will result in the generation of unmodeled dynamics in the form of changes in dynamic parameters, which will affect the controller performance. In this section, we describe the details of the complexities associated with high-rate systems.

### 3.1. High-Rate Systems-Specific Challenges

More precisely, high-rate systems are characterized by system complexities to include noise, uncertainty, unknown inputs, time varying parameters/states, and disturbances. Due to the high amplitude of events, noise can be introduced in many ways such as metal parts chattering at interfaces, circuit boards flexing, etc. There are many uncertainties in high-rate systems due to material response at high rates of loading being unknown [21] as well as uncertainties in boundary conditions. Unknown inputs are due to environmental influences that excite the system, and may include different dynamics, levels of chaos, and amplitudes for a single system. High-rate systems normally contain some time varying component that results from damage or changes in mechanical configuration from deformation of parts. In addition, disturbances may be present from the turbulent nature of the system which may excite resonances at the system or sensor levels. The challenges to making accurate estimations in the presence of these system complexities are briefly discussed in what follows. Conventional estimators fail at quickly estimating states when noise and uncertainties are present [22]. In practical applications, noise may arise from sensor measurements, algorithm implementation, contaminating estimation values, etc. The KF can be used to suppress noise if the noise function is appropriately built into its architecture. This noise suppression comes with added computational costs. To reduce the convergence time, the observer gain may be increased. This, however, can negatively impact the precision of the estimation as the noise can be amplified. One solution is a Uniform Robust Exact Observer (UREO) as proposed in [23]. It is tunable, and can achieve optimal convergence while maintaining precision under measurement noise.

Uncertainty is another common issue for many practical applications. For example, a major challenge for observer-driven algorithms used to estimate reaction rates in stirred tank bioreactors is in the difficulty of modeling the growth kinetics of micro-organisms [24]. Various techniques have been researched to deal with system uncertainties. For structural damage detection, methods for the formulation of objective functions using incomplete model data have been developed [25]. Self-tuning fusion Kalman filters based on steady-state Riccati equations are demonstrated for systems with completely unknown model parameters and noise variances [26].

There are cases for which the inputs to the system are unknown due to lack of sensors or faults in the system. For these cases, special design considerations can be made to guarantee accurate state or parameter estimations. In particular, the High Gain Observer (HGO) in [27] incorporates an auxiliary-variable to estimate unknown unsteady inputs. Reference [28] describes the usefulness of an Unknown Input Observer (UIO) for making estimations when only the output to estimate state variables is available. Originally, UIOs for a plant could be designed if and only if the plant was of minimum phase. Further development of the UIO in [28] provides alternatives to the minimum phase condition. UIO for nonlinear systems with both noise and uncertainties also require further research. One possibility is presented in [22] where a robust UIO for nonlinear systems is capable of handling both noise and uncertainty.

System models containing nonlinearities other than those from the time-varying parameters are termed parameter varying nonlinear (PVNL) systems. A sufficient condition for asymptotic convergence is developed for a two degree-of-freedoms Arcak nonlinear observer in [29]. The Arcak observer form contains two observer gain matrices of which the method is extended to optimize the second observer gain. The technique is further augmented to PVNL systems using finite dimensional relaxation method for quadratic parameter dependent Linear Matrix Inequalities (LMI).

Systems operating at high-speeds may encounter unmodeled high-order dynamics which can cause resonance, also considered as disturbances. Such resonance can lead to a decrease in estimation performance and irregular accuracy. Disturbance observers (DOB), usually used in control applications, estimate a disturbance using a low-pass filter and an inverse of the nominal model. The estimated signal is used to cancel disturbances within a system which in turn makes the system robust to uncertainties and disturbances [30]. In general, high robustness is difficult to achieve using DOB because of estimation delays attributed to pole allocation. A position-acceleration integrated disturbance observer (PAIDO) was proposed as a high-performance DOB [31]. The PAIDO structure incorporates accelerometer measurements to enlarge the frequency bandwidth of the disturbance estimation to provide better robustness [32].

Another challenge is that many of the above mentioned techniques add complexities to the estimation algorithms which slow the computational time. The decrease in computational time is another big hurdle for high-rate estimation for obvious reasons. The next section will give an example of a high-rate laboratory experiment and argue that a simple fast estimator is not sufficient for high-rate systems.

### 3.2. High-Rate Dynamic Example

**Figure 1:**
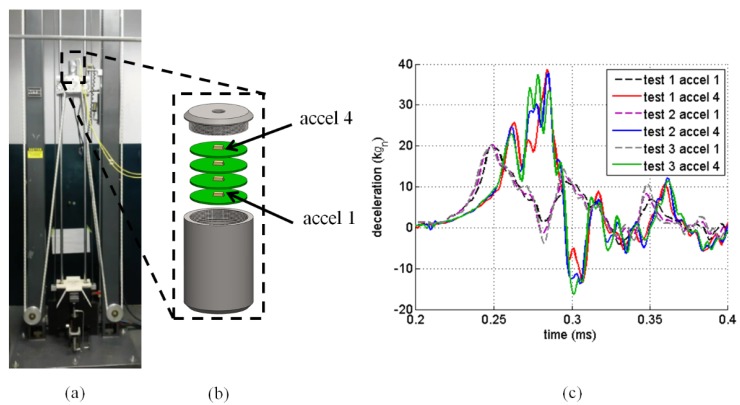
Experimental setup: (**a**) MTS-66 drop tower; (**b**) electronics unit; and (**c**) deceleration from three tests.

An example of a high rate laboratory experiment is shown in Figure 1. Figure 1a is a picture of an MTS-66 drop tower designed to generate various impact conditions. In Figure 1b is an electronics unit which consists of four circuit boards with high-g Meggitt 72 accelerometers mounted on each. These high-g accelerometers are able to accurately measure accelerations of 120,000 $g_{n}$ or 120 k$g_{n}$ [33], where 1 $g_{n}$ = 9.81 m/s${}^{2}$ = 32.2 ft/s${}^{2}$. The electronics are potted in the canister with potting material to secure all parts in place. Figure 1c shows the deceleration profile from three different tests, exhibiting varying time series behaviors for all three tests. These differences are attributed to the complex high-rate nature of the experiment.

Although the experimental setup may appear simple, this high-rate problem contains many complexities. When the drop tower is operated, the cables, which are hardwired from the accelerometers to the data acquisition system, whip violently adding noise to the measurements. Careful consideration in wires are taken to minimize the noise effect, but noise cannot be eliminated. The metal interfaces rattle upon large impacts, also adding noise. The uncertainties in this example include the unknown high-rate response of the circuit board and potting materials, and the unknown changing boundary conditions such as the bond of the potting material to the interior of the canister. The precise input to the system resulting from the drop tower impact is unknown. Disturbances created from sensor and/or system resonance is not obvious in these tests, however, it is not uncommon in larger impact tests.

The deceleration event in drop tower tests typically lasts for 0.5 ms. Due to the short duration of high-rate events, fast estimators are required. Furthermore, the estimator must be capable of handling system complexities mentioned above. In contrast, examples of fast estimators can be seen in the sensorless control of induction motors or alternating current (AC) motors, commonly used in industrial applications [34]. Different methods have been explored for estimating states of induction motors. Bodson et al. in [35] demonstrate precise motor controls in 73 ms using backward difference for the position measurement using low-pass filtering with a nonlinear observer constructed using a mathematical model of the system. Xu et al. in [36] show an adaptive sliding observer capable of a computation time for one update of 19 μs, and an Extended Kalman Filter (EKF) capable of 86 μs using a 250 MHz processor. Zhang et al. in [37] compare the performance of a Luenberger observer (LO), sliding mode observer (SMO), and an EKF. Using a 150 MHz processor, one update took the LO and SMO 5 μs to converge while the EKF took 100 μs. These observers demonstrate the capability of performance in the microsecond range appealing to the high-rate problem.

While the research efforts surveyed above represent important progress in state estimation, in most cases a particular state-estimator type addresses a specific system complexity challenge. As argued previously, the critical challenge with high-rate state estimation is associated with the presence of most of the discussed complexities in a single system. In the next section, a brief survey on observers is conducted to develop a foundation for a potential solution. The survey is intended to provide the reader with a broad background to understand the possible impact that the choice of an observer may have on systems experiencing high-rate dynamics.

## 4. Background on Observers and Their General Applicability

This section reviews typical families of observers in view of the applicability to the state-estimation problem. The most simplistic class of observers are those designed for linear and idealized systems. Such observers have, typically, fast computation time and convergence due to the triviality of computations. The Luenberger Observer (LO) [38] is a classic observer used for linear systems with well-defined numerical models. The LO has proven itself to be valuable in the areas of system monitoring and regulations, detecting, and identifying failures in dynamic systems [39]. However, since the LO is heavily dependent on the mathematical model of the system, disturbances, dynamic uncertainties, and nonlinearities can be particularly challenging. The KF [40] can be used in linear applications where noise is present and characterized as Gaussian [36]. The reason for the word “filter” stems from the fact that the algorithm filters through noisy data to converge to accurate estimations. While the KF typically produces estimations with higher accuracy than the LO, its implementation is more complex [37] due to possibly unmodeled nonlinearities in the system model and ill-conditioning of the covariance matrix [41]. The Sliding Mode Observer (SMO) [42] can be an alternative to provide very good robustness [43,44,45,46,47], but is sensitive to the choice of gain [48]. The SMO exhibits ripples in the presence of external noise [37].

Tuning of observers for linear systems can be conducted through the pole placement method. The pole placement method allows the tuner to determine observer gain matrix values based on the desired eigenvalues for system stability and convergence. The preferred location of the eigenvalues depends on the application. Generally, the further to the left in the complex plane or the more negative the real part of the pole, the faster the convergence rate. Placing the poles too far left may amplify noise [49]. The LO, KF, and SMO may exhibit high convergence rates for linear systems. However, the majority of practical problems are nonlinear and complex. For this reason, observers built for nonlinear systems are more appropriate for applications to the high-rate state estimation problem. They are discussed in what follows.

### Observers for Nonlinear Systems

Observers for nonlinear systems can be classified into three main estimation methods: data-driven, statistical, and model-driven methods [50]. Popular data-driven methods include nonlinear autoregressive moving average (NARMAX) models [51], fuzzy logic [52] and neural network (NN) estimators [53]. The performance of data-driven method is linked to the quality of data mining and interpretation algorithms, and an additional limitation can be found in the computational time required to achieve an appropriate estimate [54]. Data-driven methods were developed as tools to process information without knowledge of a system’s dynamics, particularly useful for handling very complex systems. For instance, Geetha et al. [55] compared the performance of an NN and an Extended Kalman Filter (EKF) based state filter for the application to a Continuous Stirred Tank Reactor (CSTR). The performance metric indicated that the NN displayed smaller errors in the estimations over a unit sampling time than the EKF, because the CSTR had complex, nonlinear dynamics, which are difficult to characterize.

Statistical methods include Least Squares Estimator (LSE) [56], Maximum Likelihood Estimator (MLE) [57], and Particle Filters [58]. The advantage of statistical methods is the probabilistic prediction capabilities based on known parameters. These techniques can be used for nonlinear models with non-normal data. They bypass the need for linearized dynamic equations allowing global convergence of estimations. Statistical methods with simple properties such as the Least Squares method are popular in control, signal processing, and prediction theory applications. For instance, Ortega [59] discusses a method for reducing an online Least Squares parameter estimator to a set of regression vectors which guarantees a finite convergence time. The weakness of this approach is the algorithm’s extreme sensitivity to noise. Franzho and Sherman [60] introduced a minimum variance spectral estimator, also known as Capon’s maximum likelihood spectral estimator, that converges to a signal point spectrum even with no knowledge of the noise spectral characteristics, making it robust to contaminating noise types. Bai et al. in [61] proposed a robust statistical estimator that takes an analytic center approach for bounded error parameter estimation. The analytic center estimate minimizes the logarithmic average output error and can be implemented in a sequential form. This method comes with increased computational complexity. In [62], a Modified Recursive Least Squares estimation technique was developed for adaptive control applications were the weaker sufficiently exciting [63] condition is more likely to be satisfied than the stronger persistently exciting [64] condition. A general limitation of statistical methods is the reliance on available data sets for training and/or extraction of probability distribution functions.

Model-driven methods include the EKF, Unscented Kalman Filter (UKF), variations of the SMO, HGO, Nonlinear Extended State Observer (NESO), Robust State Estimators (RSE), and many more. The EKF uses a linear approximation of the nonlinear system [65] either by taking the derivative of the nonlinear function or by applying a Taylor series expansion to the desired order of the approximation. Depending on the order of the approximation, the observer is termed reduced, full, or higher order [41]. A challenge with the EKF is the linearization of the system making the corresponding propagation equations available only to the neighborhood of the estimate [66]. The EKF can be difficult to implement, difficult to tune, and highly unstable unless the system is nearly linear on the time scales of the update calculations [67]. It requires longer time for convergence compared with the LO and SMO as its computations are more complex [37]. The EKF is also difficult to apply to nonlinear systems with time varying parameters, particularly in the presence of noise [24]. The UKF uses the true nonlinear model and approximates a Gaussian distribution of the state random variable. The UKF avoids the use of complex Jacobian and Hessian matrices, making it easier to implement than the EKF with simpler computations [68]. Crassidis and Markley [69] demonstrated the superiority of the UKF with respect to the EKF in terms of accuracy, computational costs, and ease of implementation. Charles et al. [70] discussed the Utkin and Walcottzak variations of the SMO observers, both being robust estimators. However, both variations of the SMO underperform in the estimation of states when some inputs are unavailable. HGO uses a sufficiently high observer gain that will guarantee good performance of the observer in terms of accuracy and speed of convergence [71]. For most cases, the HGO is used as an LO-type estimator with large gain and the application is for estimating slowly varying states or inputs [27]. A disadvantage of the HGO is strong chattering when the gain is very large [72]. The NESO actively estimates the states, uncertainty, and unknown disturbances even when the system is unknown [29,73]. The RSE guarantees robustness of the designed estimator if time invariant nominal system matrices, constant filter design parameters, and stationary external inputs conditions are met. The computational complexity is comparable with that of the KFs [74].

There are other model-driven observers used to estimate nonlinear systems. To name a few, in [75], an SMO was implemented on a system that was linearized using global linearization. Ticlea and Besancon [76] discussed an immersion-based observer design where the dimension of the state is increased beyond what is done in the ESO, in order to obtain a new representation better suited for observer design. The Fokker-Planck Equations (FPE) are used to transform space and time fractional partial differential equations to a system of ordinary differential equations that are more easily solved [77], which could reduce computational time. Daum in [78] designed an exact nonlinear recursive filter where instead of linearizing nonlinear equations as conducted with the EKF, the filter solves a special class of nonlinear problems exactly with comparable computation complexity to the EKF. The coordinate transformations with output injections method is studied in [79,80,81,82]. The authors in [83] propose a dynamic observer using the Moore-Penrose generalized matrix inverse of the state matrix. The model-driven estimation method has attracted much attention because it can produce accurate state estimations when it is not possible or practical to have sensors to characterize every state [84]. Furthermore, the mathematical models required for control purposes are readily available [85]. Table 1 summarizes the observers discussed in this section in terms of general applicability to the problem of high-rate state estimation.

## 5. Adaptive Observers

A useful tool to cope with the problem of system complexities are adaptive observers (AOs). AOs can be used to estimate states and parameters using input-output measurements, ideal for handling uncertainty in state estimation [86]. They are typically characterized by asymptotic stability but slow convergence rates [87]. These observers are often modifications of the observers discussed in Section 4 that use linear transformations, such as the LO, SMO, and versions of the KF. In particular, adaptive observers have been proposed to estimate the unmeasurable states for different classes of nonlinear systems [88].

Adaptive versions of the HGO and the EKF have been studied in [89]. The high gain aspect of the HGO serves to eliminate the nonlinear part of the system, while allowing rapid convergence, thus making it a good candidate for nonlinear systems. On the other hand, the EKF filters out system or process noise, therefore advantageous for field applications. The two were combined to create a high-gain extended Kalman filter (HG-EKF) [89], which possesses the advantages of both estimators. The authors showed that the resulting HG-EKF could converge at a desired speed with the tuning of only one parameter while also being capable of noise rejection. Although promising, a key issue with the HG-EKF is with the exponential convergence occurring only at the beginning of the estimation process from the initial condition. Large perturbations in the system are difficult to estimate if they are not already modeled within the system. This issue was overcome with the development of the adaptive gain extended Kalman filter (AG-EKF) [89]. The main advantages of this observer are the noise rejection and the ability to estimate perturbations. The trade-off is that the convergence rate of the AG-EKF is slower than the HGO or HG-EKF due to the algorithm’s complexity.

While showing great promise, AOs are known to have slower convergence rate. In related studies, Shahrokhi and Morari [87] attributed this problem to the utilization of single observation errors. An arbitrarily fast convergence rate is achieved with the use of a discrete identifier which uses multiple output errors. Global asymptotic stability is assured for sufficiently rich inputs. This method is shown to be robust and insensitive to input types and initial conditions. Khaytati and Zhu [88] identifies that complicated adaptation laws are the cause of slow convergence. As a solution, an adaptive observer with exponential parameter estimation dynamics for nonlinear systems with unmeasured regression terms is introduced, requiring Lipschitz and bounded nonlinearity constraints. In this AO configuration, a parameter can be selected as large enough to increase the rate of convergence. However, if the value is too large, it will attenuate the gain matrix. Rajamani [90] shows that the stability of the system is not based on eigenvalues alone. The eigenvalues have to be located sufficiently far left in the complex plane and the eigenvectors need to be sufficiently well-conditioned. This condition is limited in the sense that the Lipschitz constant of the nonlinear part has to be small. To obtain less conservative results, a generalized Lipschitz condition is used in conjunction with an adaptive observer for a wider class of nonlinear systems [3]. Byrski and Byrski [91] propose a modification of a modulating functions method (MFM) which enables rapid identification of step changes to parameters with minimal time delays in the estimations. There are several different solutions to increasing the rate of convergence for adaptive observers as potential candidates to high-rate estimation problems as previously discussed and summarized in Table 2.

Model-driven methods have the advantage of providing precise measures of damage due to the availability of models, therefore enabling condition assessment and system prognosis. However, they require knowledge of the physical model, which is a difficult task for real-world systems. Additionally, high-rate systems may experience changes in the structure requiring different model parameters than initially specified. Statistical methods can identify faults through a probabilistic measure, and may be used to conduct prognosis by evaluating the probability of faults, but require knowledge of probability distribution functions. The statistical properties are usually calculated from numerous tests, which is difficult to achieve for high-rate systems. Data-driven methods, in general, can provide accurate estimations based on pattern recognition and classification. Alternatively, they require precise examples and extensive training over available data set. Due to the spontaneous occurrence of high-rate events, little insight is provided in the external loads and system changes. Model- and statistics-driven methods will require significant developments to be applicable to high-rate systems.

Adaptive data-driven methods, on the other hand, can be seen as black-box systems capable of handling uncertainties found in state estimation [86]. These methods were developed as tools to process information without any knowledge of a system’s dynamics. This makes data-driven methods particularly useful for dealing with very complex systems. Geetha et al. in [55] compared an NN estimator against an EKF-based state filter for continuous stirred tank reactors. The results of the study indicated smaller errors in the estimations over a unit sampling time with the use of NN estimators over the EKF due to the complex nonlinear dynamics difficult to characterize. DeCruyenaere et al. in [92] showed that data-driven neural networks were generally capable of outperforming KFs particularly when the system includes nonlinearities or non-Gaussian process noise. Mosavi in [93] showed that, while Kalman filters had higher accuracy in global positioning systems, recursive neural networks offered overall better performance due to their faster computations times.

Hybrid approaches also count advantages by combining strengths in model- and data-driven techniques. A model-driven approach can supplement data-driven methods by accounting for already understood dynamics. Conversely, data-driven methods can supplement a model-driven observer by approximating complex or difficult-to-model processes. For example, Psichogios et al. in [94] used a neural network to capture the complex, nonlinear growth rate of bacteria in a bioreactor that could then be used in a first-principles system model to find the overall biomass concentration. Hu et al. in [95] used a multiscale framework approach with EKF to make state-of-charge and capacity estimation. These examples demonstrate the needs for data-driven methods for complex systems. This characteristic of the adaptive data-driven method may be particularly useful for high-rate systems, which face similar challenges.

## 6. Conclusions

High-rate state estimation is a challenging task especially for complex engineering systems requiring real-time observability to ensure adequate performance. Example applications discussed civil structures exposed to blast, automotive safety systems, and space shuttle and aerial vehicles prone to in-flight anomalies. These examples highlighted the high potential of high-rate state estimators to improve the resiliency of high-rate engineering systems and save lives. The paper presented a survey of existing applications and methods for state estimation that could be useful in solving the high-rate state estimation problem.

The specific challenges associated with the high-rate problem were presented. They include large uncertainties on external loads, high levels of non-stationarities and heavy disturbances, and generations of unmodeled dynamics from mechanical changes. An induction motor study was presented to make a distinction between high-rate systems and fast systems. Additionally, a variety of observers developed to compensate for system complexities of noise, uncertainty, unknown inputs, time varying parameters/states, and disturbances to improve on performance were discussed. However, these observers do not address the combination of these challenges which forms the high-rate problem.

In providing a discussion on the suitability of various observers, the strengths and weaknesses of various methods were introduced at a broad level. Three main categories of observers were reviewed: data-driven, statistical-driven, and model-driven observers. Generally, data-driven observers are advantageous when the complexity of a system does not allow for an accurate physical representation. Statistical methods perform well when prior data is available to produce a good understanding of the statistical properties of the system’s behavior. Model-driven observers produce fast and accurate estimations for systems with well defined models. The discussion extended to adaptive versions of these observers, termed adaptive observers (AOs). It was argued that given the complex nature of high-rate systems, data-driven observers have a particular promise because of the difficulty with creating a representation, and that specifically their adaptive form can be leveraged to adapt to large levels of uncertainties and complexities. However, AOs are characterized by slow convergence. Work addressing AOs’ convergence has been reviewed, and it was concluded that work remains to be done in producing fast AOs.

Of particular interest is the utilization of AOs in hybrid configurations, which may take advantage of prior knowledge on a system for improved performance. They can be leveraged to replace dynamics too complex to model with a data-driven approach, possibly leading to significantly improved computational time. It follows from the discussion presented in this paper that adaptive observers and hybrid observers offer a path to developing high-rate observers capable of microsecond estimation.

## Figures and Tables

**Table 1:** Summary of observers in terms of general applicability to the problem of high-rate state estimation.

Observer Type	Application to High-Rate State Estimation	Reference
Luenberger Observer (LO)	Very fast convergence rates, but generally applies to linear systems with precise nominal models, thus inadequate for high-rate problem.	[39]
Sliding Mode Observer (SMO)	High robustness and improved results for inaccurate models, but sensitive to choice of gain limiting the convergence rate.	[43,44,45,46,47,48]
Extended Kalman Filter (EKF)	High accuracy for nonlinear systems with added noise, but complex implementation leading to poor convergence rates.	[37,41]
Unscented Kalman Filter (UKF)	Better convergence rates and higher accuracy than the EKF for it uses true nonlinear model, avoids complex Jacobian and Hessian matrices, and is easier to implement.	[68]
High-Gain Observer (HGO)	Accurate and fast convergence rates for estimating slowly varying states or inputs making it inadequate for high-rate problems.	[27,71]
Nonlinear Extended State Observer (NESO)	Offers robustness to system uncertainty and external disturbances. Outperformed both HGO and SMO in a comparative study.	[39,73]
Robust State Estimator (RSE)	Guarantees robustness for time invariant systems, constant filter design parameters, and stationary external inputs, however the convergence rate is similar to Kalman Filters.	[74]

**Table 2:** Adaptive observer addressed challenges and solutions to increase convergence rates.

Addressed Challenge	Solution	Reference
Sensitivity to noise	HG-EKF	[89]
Sensitivity to large perturbations	AG-EKF	
Arbitrary fast convergence	Using multiple output errors	[87]
Adaptation laws	Exponential parameter estimation	[88]
Broad applicability	Generalized Lipschitz condition	[3]
Fast identification of step changes	MFM	[91]
